# 3D Vision-Guided Adaptive 3D Ultrasonic Scanning for Robotic Arms: Nondestructive Testing of Aerospace Components

**DOI:** 10.3390/s26072129

**Published:** 2026-03-30

**Authors:** Xiaolong Wei, Zijian Kang, Yizhen Yin, Jingtao Zhang, Caizhi Li, Yu Cai, Weifeng He

**Affiliations:** 1 National Key Lab of Aerospace Power System and Plasma Technology, Air Force Engineering University, Xi’an 710038, China; weixiaolong9s@126.com (X.W.); 18703092003@163.com (Z.K.); kbzjt913@163.com (J.Z.); hehe_coco@163.com (W.H.); 2 Institute of Aero-Engine, School of Mechanical Engineering, Xi’an Jiaotong University, Xi’an 710038, China; yinyizhen@stu.xjtu.edu.cn

**Keywords:** aircraft composite, laminated structures, 3D ultrasonic testing, deep learning, vision guidance, point cloud processing

## Abstract

In view of the bottleneck problems existing in the 3D ultrasonic testing of aircraft composite laminated structures—including heavy reliance on manual operation, resulting in low detection efficiency, and the inability of traditional robotic arms to adapt to the testing of complex curved surfaces due to their dependence on predefined fixed trajectories—this paper proposes an automated 3D ultrasonic testing method based on 3D vision guidance for robotic arms. Firstly, the proposed Yolo-Mask model is adopted to realize the visual recognition and segmentation of composite component regions, after which the segmentation results are mapped to the depth map and further converted into the surface point cloud of the material. Secondly, on the basis of point cloud preprocessing and trajectory point extraction, the automatic planning of the robotic arm’s scanning trajectory is achieved, which drives the robotic arm to perform precise motion and to synchronously collect spatial pose and ultrasonic testing data. Finally, 3D reconstruction is completed via a fusion algorithm, and 3D images of the material’s internal structures are generated. Experimental verification shows that the proposed method achieves a Segm-mAP of 97.4%, a detection speed of 11.7 fps, and a 3D imaging error of less than 0.1 mm, thereby realizing fully automated detection throughout the entire process. This research provides an effective solution for the non-destructive testing of aircraft composite structures.

## 1. Introduction

To ensure the geometric correctness of imaging, undistorted ultrasonic images, quantifiable measurement and reproducible diagnostic results, real-time acquisition of the spatial position and angular orientation information of the ultrasonic probe is an essential requirement during the ultrasonic testing process. Roller-type position encoders are commonly adopted for this purpose in the industry. They feature a simple structural design, stable response performance and controllable manufacturing costs. However, in practical applications, slippage is liable to occur between the rollers and the tested surface, making such encoders unable to operate reliably on 3D curved surfaces. Furthermore, their heavy dependence on manual operation leads to limited automation, which fails to meet the demands for high-efficiency non-destructive testing [1,2].

By contrast, multi-axis CNC scanning systems serve as a superior approach for high-precision position encoding [3,4]. CNC stands for Computer Numerical Control, which refers to computer numerical control technology. Such systems fundamentally eliminate the occurrence of slippage and provide direct positional feedback through high-precision linear encoders or servo encoders, thereby enabling fully automated and repeatable scanning along predefined paths. Nevertheless, multi-axis CNC scanning systems are associated with high manufacturing costs and limited motion degrees of freedom; their insufficient flexibility in the face of complex free-form surfaces renders them unfit for direct deployment in field testing applications.

To overcome the inherent limitations of roller-type position encoders and multi-axis CNC scanning systems, 6-degree-of-freedom (6-DOF) robotic arms offer a more advanced technical solution. Their encoding mechanism is fundamentally distinct from that of the two aforementioned methods: the spatial pose is not measured directly but is derived via the forward kinematic solution of the robotic kinematics model based on the real-time readout values of high-precision rotary encoders installed on each joint of the robotic arm. This indirect encoding method endows 6-DOF robotic arms with excellent motion flexibility, allowing the ultrasonic probe to perform high-precision scanning on various complex curved surfaces adaptively.

At present, extensive research has been carried out domestically and internationally on the control of ultrasonic probes for scanning operations via robotic arms [5,6,7,8]. Zang et al. [9] developed a multi-degree-of-freedom (multi-DOF) robotic arm named UTArm for curved surface testing, which realized lightweight structural design via topological optimization and completed the establishment of kinematic and dynamic models, thus providing a professional solution with high precision and low cost for ultrasonic non-destructive testing. Li et al. [10] systematically elaborated the technical system of autonomous robotic ultrasonic data acquisition and proposed a standardized autonomy grading framework encompassing key technologies such as scanning path planning, contact force control and ultrasonic image quality optimization. Bao et al. [11] developed an ultrasonic robotic system integrated with force/torque measurement and control functions, which is capable of real-time monitoring and maintaining a constant contact force, and achieved a force control accuracy of 0.57 N in experimental tests.

Deng et al. [12] proposed a vision-guided robotic arm ultrasonic scanning method, which fused multi-view point clouds with skeletal recognition to reconstruct human body surfaces, planned scanning paths via point cloud slicing, and introduced ultrasonic image feedback to adjust the probe pose, ultimately validating the feasibility of the method through human-subject experiments. Deng et al. [13] developed a portable robotic ultrasonic system for follow-up diagnosis, which adopted a 6-RSU parallel mechanism and was integrated with a force control algorithm and multi-modal reinforcement learning. This system realized the automatic acquisition of multi-target ultrasonic images and obtained imaging performance with a Structural Similarity Index Measure (SSIM) of 0.764 and a Normalized Cross-Correlation (NCC) of 0.977 in experiments. Zhao et al. [14] proposed a vision-guided robotic arm-assisted ultrasonic phased array testing method, which realized the non-destructive testing of welded components via point cloud matching and path planning, with a defect quantification error of only 6%. Ma et al. [15] proposed a robot control method based on 3D scanning, which automatically generated raster scanning paths from 3D point cloud data and enabled laser ultrasonic testing of curved composite structures without the need for pre-defined geometric models.

Most of the aforementioned intelligent ultrasonic testing systems based on robotic arms are highly dependent on the input of visual information to complete the planning and guidance of robotic arm operation paths. At present, single-target tracking algorithms suitable for ultrasonic probes mainly fall into three categories: first, traditional filter-based methods (e.g., KCF [16], which stands for Kernelized Correlation Filters, a classic target tracking algorithm based on correlation filtering), which offer high computational efficiency but suffer from limited robustness in complex scenarios; second, detection-based tracking frameworks (e.g., Atom [17], Siam R-CNN [18] and PrDiMP [19]), which typically exhibit strong discriminative ability and anti-interference performance yet are constrained by low operating speed, making them difficult to apply to real-time probe tracking tasks; and third, Siamese network-based tracking methods (e.g., SiamFC [20], SiamRPN [21], SiamRPN++ [22], SiamMask [23] and SiamFCA [24]), which possess the dual advantages of high tracking accuracy and fast inference speed.

In addition, research based on binocular cameras has also made certain progress. Cygan et al. [25] developed a low-cost 3D visual positioning system based on binocular cameras, which achieves ultrasonic probe positioning through marker point recognition and triangulation, with a verified positioning error of no more than 0.7 mm. Zhu et al. [26] proposed a vision-compensated ultrasonic imaging method that dispenses with position encoders; based on binocular cameras, this method achieved a detection accuracy of 94% and real-time probe tracking at 21 fps on an improved YOLOv5s model. Therefore, the current optical detection and pose perception technologies for ultrasonic probes have achieved a high level of accuracy.

Based on the above analysis, it can be concluded that the current robotic arm ultrasonic scanning technology has formed a relatively complete technical system in the aspects of specialized robotic arm design, systematic technical frameworks, high-precision force control, and vision-guided scanning. However, the existing applied research for industrial testing, in particular for aircraft composite laminated structures, remains insufficient [27,28,29,30], and the scanning performance of such technologies on actual aerospace components still awaits further experimental verification. In addition, there is still room for improvement in terms of spatial perception fusion and the balance of scanning accuracy.

To address the above issues, this paper conducts research on an intelligent ultrasonic testing method based on 3D vision-guided robotic arms for aircraft composite laminated structures. By fusing 3D visual perception and adaptive path planning, the method achieves precise control of the probe pose and autonomous optimization of the scanning trajectory without the need for pre-defined geometric models, aiming to improve the scanning coverage quality and detection efficiency of components with complex curved surfaces.

The specific innovations of this paper are as follows:

1. The advanced region segmentation algorithm is used to identify and divide the composite components; the segmentation results are mapped to the depth map, and the point cloud of the material surface is obtained by transformation.
2. The surface point cloud of the material is used to carry out voxel downsampling, normal vector calculation, isospace adoption and trajectory point extraction, and the manipulator is commanded to carry out precise motion according to the extracted trajectory points.
3. The proposed fusion algorithm reconstructs the spatial coordinates and ultrasonic information in three dimensions to generate a three-dimensional image of the material’s internal structure, thereby enabling precise spatial localization of damage and automated assessment of structural integrity.

## 2. Pre-Experiment Preparation

### 2.1. Construction of Experimental Platform and Dataset

**Figure 1:**
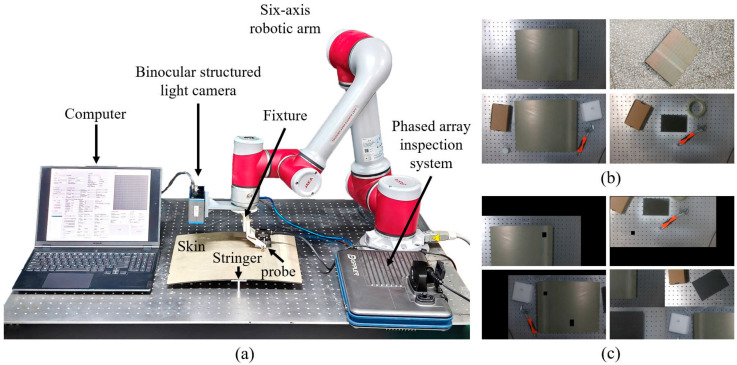
Experimental platform and dataset for robotic arm vision-guided ultrasonic automated inspection: (**a**) Experimental platform, (**b**) visible light dataset, (**c**) data augmentation.

Figure 1 shows the schematic diagram of the experimental platform and dataset for the vision-guided robotic arm ultrasonic automatic inspection system proposed in this paper. Figure 1a presents the experimental platform for the aforementioned inspection system, which mainly consists of a six-axis robotic arm, an ultrasonic testing probe, an industrial 3D camera, and an end tooling bracket. Specifically, a Jaka collaborative robotic arm (Model: Jaka Zu7 Manufacturer: JAKA Robotics Co., Ltd.) is adopted as the six-axis robotic arm, which serves to drive the industrial 3D camera and ultrasonic probe to perform translational and rotational movements. The industrial 3D camera is a SurfaceHD50 model produced by GeniSense, which is used to acquire visible light images and depth maps of test pieces. The ultrasonic probe is a phased array probe manufactured by Doppler and is dedicated to conducting damage detection on composite materials.

The specific details of the ultrasonic probe used in the experiment are as follows:

1. Number of elements: The phased array ultrasonic probe (Doppler) used in the experiment has 64 elements, arranged in a linear array.
2. Central frequency: 5 MHz, which is suitable for damage detection of composite laminates with a thickness of 2–20 mm.
3. Signal digitization method: The ultrasonic signals collected by the probe are transmitted to a dedicated ultrasonic acquisition card (sampling rate: 100 MHz, resolution: 16 bits) for analog-to-digital conversion. The acquisition card is synchronized with the robotic arm’s pose data acquisition system to ensure time–space consistency between the ultrasonic signals and the probe’s spatial position.

The end tooling fixture is independently designed for this research; it was modeled and drafted using SolidWorks 2021SP5 and fabricated via 3D printing or precision machining processes according to actual requirements. This fixture functions to rigidly fix the industrial 3D camera and the ultrasonic probe to the end-effector of the six-axis robotic arm.

Figure 1b,c illustrates the construction process of the segmentation dataset for composite laminated structures. Data on various composite laminated structures were acquired using a structured light camera, with the placement positions of the structures randomly altered. As the shooting pose of the industrial 3D camera is fixed directly in front of the composite laminated structure during the robotic arm’s vision-guided probe scanning process, the shooting angles of the camera were also focused on multiple viewing angles directly in front of the composite laminated structures during the data acquisition phase. This setup ensures data applicability and enhances data diversity, and a total of 800 original images were collected in this experiment.

After the completion of data acquisition, LabelMe 5.4.0 was adopted for data annotation. Then, at the original image level, the 800 original images were first divided into training (640 images), validation (80 images) and test sets (80 images) at a ratio of 8:1:1. Subsequently, four typical data augmentation methods, namely flipping, random cropping, noise addition and Mosaic, were applied to each subset independently to generate 4000 images in total. The final dataset, consisting of 4000 images after augmentation, was partitioned into the training set, test set and validation set at a ratio of 8:1:1 for the subsequent experiments.

### 2.2. Calibration of the Robotic Arm Coordinate System

**Figure 2:**
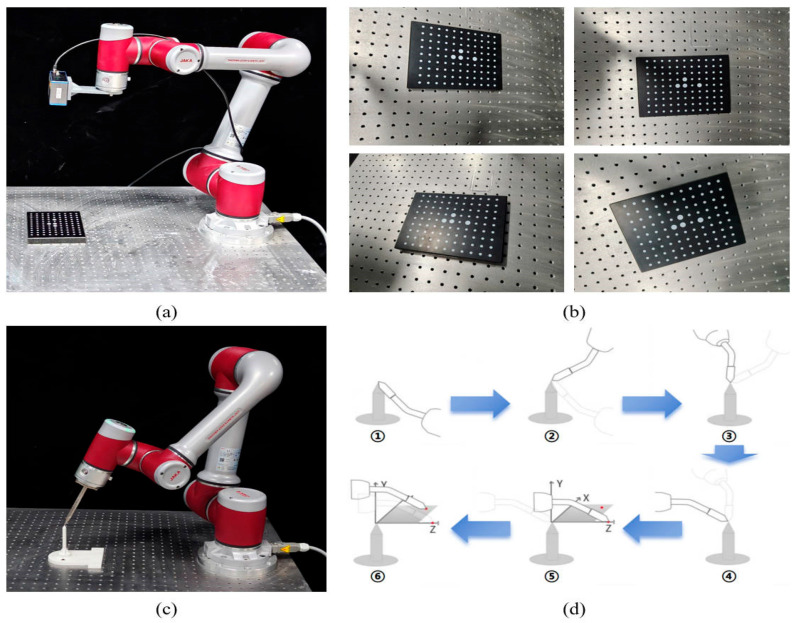
Processes of hand-eye calibration and TCP calibration: (**a**) Data acquisition for hand-eye calibration, (**b**) partial hand-eye calibration data, (**c**) TCP calibration operation, (**d**) six-point method for TCP calibration process.

Calibration of the robotic arm coordinate system constitutes a core prerequisite for the implementation of vision-guided operations. Since the vision system, robotic arm body and end-effector each have an independent coordinate system, only by accurately calibrating and establishing the transformation relationship between them can the position and pose of the test piece captured by the camera be precisely converted into motion commands executable by the robotic arm, thereby forming a closed-loop control system. This calibration process specifically includes hand-eye calibration [31] and TCP calibration [32]. Figure 2 illustrates the processes of hand-eye calibration and TCP calibration.

Robotic hand-eye calibration refers to calibrating the transformation relationship between the camera coordinate system and the robotic arm coordinate system, and the eye-in-hand mode is adopted in this paper for calibration. As shown in Figure 2a,b, by calling the hand-eye calibration interface in the secondary development kit of the industrial camera, the robotic arm is controlled to change its shooting pose more than twenty times as required, and a customized circular dot calibration board is photographed at different positions. The pose information of the corner points on the calibration board and the pose information of the robotic arm’s wrist joint at the current shooting pose are recorded, and finally, the calibration script is run to obtain the calibration results. To quantitatively evaluate the hand-eye calibration performance, we will calculate and report the reprojection error (pixel-level) and AX = XB equation residual error (mm-level) based on the calibration data, and conduct 20 repeated calibration tests to obtain the mean and standard deviation of the errors, reflecting the stability of the hand-eye calibration method.

Tool Center Point (TCP) calibration refers to calibrating the positional relationship between the robotic arm’s wrist joint coordinate system and the tool center point of the end-effector. As shown in Figure 2c,d, TCP calibration is performed using the six-point method by fabricating an equivalent sharp-point tool, and the obtained results are the rotation matrix and the translation matrix between the robotic arm’s wrist joint coordinate system and the tool center point. The specific steps are as follows: select a fixed sharp tip in the workspace, use the teach pendant to control the acting point of the metal cone at the robotic arm’s end-effector to coincide with the fixed sharp tip at different poses, collect four sets of position data and record the pose of the robotic arm’s end-effector at these positions; then control the grinding tool at the robotic arm’s end-effector to move a certain distance along the positive directions of the X and Z axes, record the robotic arm’s pose at these two positions; and finally obtain the transformation matrix of the tool coordinate system relative to the central coordinate system of the end flange of the robotic arm. For TCP calibration, we will report the position repeatability error and the attitude repeatability error of the tool center point and conduct 20 repeated calibration tests to obtain the mean and standard deviation of the errors, reflecting the stability of the TCP calibration method.

Furthermore, we will establish a complete calibration uncertainty budget: decompose the uncertainty sources of the entire calibration chain (including camera intrinsic parameter calibration uncertainty, hand-eye calibration pose sampling uncertainty, and TCP calibration operation uncertainty), quantify the uncertainty value of each source, and use the error propagation law to calculate the combined standard uncertainty of the calibration result. Based on the chained transformation relationship of the vision system–robotic arm–end effector, we will also supplement the calibration uncertainty propagation analysis to 3D localization and dimensional errors: establish the mathematical model of calibration uncertainty propagation, quantitatively analyze the contribution of calibration uncertainty to the final 3D reconstruction positioning error and dimensional measurement error, and add the corresponding error propagation curve and quantitative results in the manuscript.

## 3. Methods

**Figure 3:**
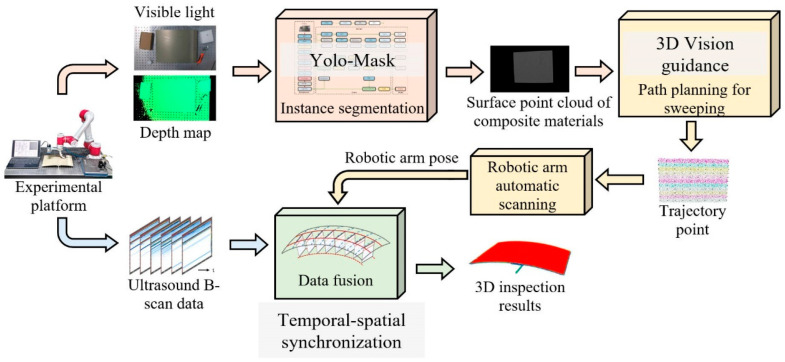
The structure of robotic arm 3D vision-guided system.

The intelligent ultrasonic testing method based on 3D vision-guided robotic arms proposed in this paper enables the intelligent planning of robotic arm scanning trajectories and 3D ultrasonic testing of the internal structures of materials. Figure 3 illustrates the overall framework of this method, which mainly consists of three core components: a Yolo-Mask-based instance segmentation network for composite material surfaces, a 3D vision-guided robotic arm ultrasonic scanning method, and a fusion method for robotic arm pose and ultrasonic data.

Its main steps are as follows: (1) Driven by the robotic arm, the binocular structured light camera on the experimental platform captures images of the composite test piece to acquire the visible light images and depth maps of the composite test piece to be inspected. (2) An instance segmentation network for composite material surfaces based on Yolo-Mask is designed to perform instance segmentation on the composite material surface regions in the visible light images. The segmented regions are mapped to the depth maps, and then the composite material surface point cloud is calculated and derived based on the depth maps within the segmented regions. (3) A 3D vision-guided ultrasonic scanning method for robotic arms is designed to conduct a series of calculations on the composite material surface point cloud, including downsampling processing, normal vector calculation, region division and selection of detection path trajectory points, so as to obtain a set of motion trajectory points. (4) In combination with the pose transformation matrix relationship obtained from the calibration of the coordinate system, the six-dimensional pose coordinates of the trajectory points of the ultrasonic testing path are calculated. After transformation into the robotic arm coordinate system, the robotic arm drives the ultrasonic probe to complete the ultrasonic scanning of the composite laminated structure in accordance with the selected detection path trajectory and acquires continuous ultrasonic B-scan data simultaneously during the scanning process. (5) A fusion method for robotic arm pose and ultrasonic data is designed, which calculates the pose of ultrasonic probe elements based on the robotic arm pose, fuses the processed ultrasonic B-scan data with the probe element pose information through spatiotemporal synchronization to obtain 3D ultrasonic point cloud data, and generates 3D images of damage in composite material structures after the visualization of the 3D ultrasonic point cloud data.

### 3.1. Yolo-Mask-Based Instance Segmentation Network for Composite Material Surfaces

#### 3.1.1. Yolo-Mask Network Design

Images of composite test pieces directly captured by the structured light camera also contain interference from the background and foreign objects in addition to the material itself. Since only the 3D point cloud data of the material surface is required for robotic arm scanning path planning, a Yolo-Mask instance segmentation network for composite material surfaces is designed in this paper to realize the instance segmentation of composite material surfaces in visible light images. As there is a one-to-one correspondence between visible light images and depth maps, mapping the segmented regions to the depth maps enables the instance segmentation of composite material surfaces in the depth maps.

**Figure 4:**
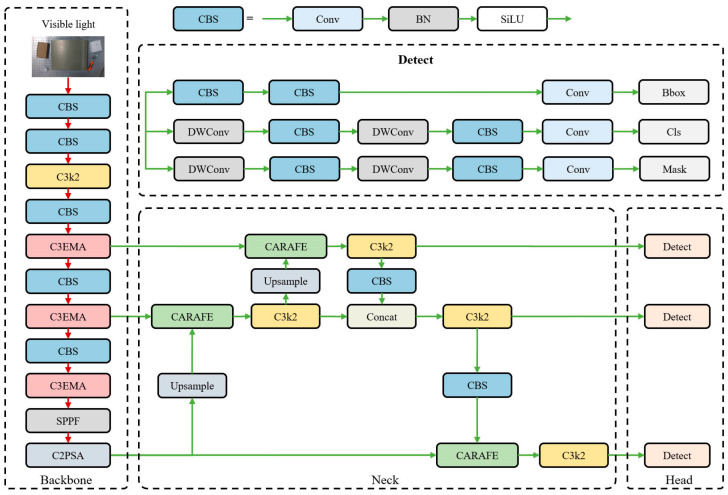
Yolo-Mask network.

Yolo-Mask is a model optimized in multiple dimensions for object detection and instance segmentation tasks on the basis of the Yolo11 framework. Its core improvements cover two key components, namely the Backbone and the Neck. By introducing an advanced attention mechanism and upsampling strategy, the model’s performance in multi-scale feature extraction, context information fusion and mask prediction is further enhanced. In the Backbone component, the Efficient Multi-Scale Attention (EMA) [33] module is introduced into the C3k2 module to replace it with the C3EMA module. In the Neck component, the Content-Aware ReAssembly of FEatures (CARAFE) [34] module is incorporated to replace the original upsampling module. The specific structure of the finally improved model is shown in Figure 4.

#### 3.1.2. Network Structure Optimization Method

- C3EMA

**Figure 5:**
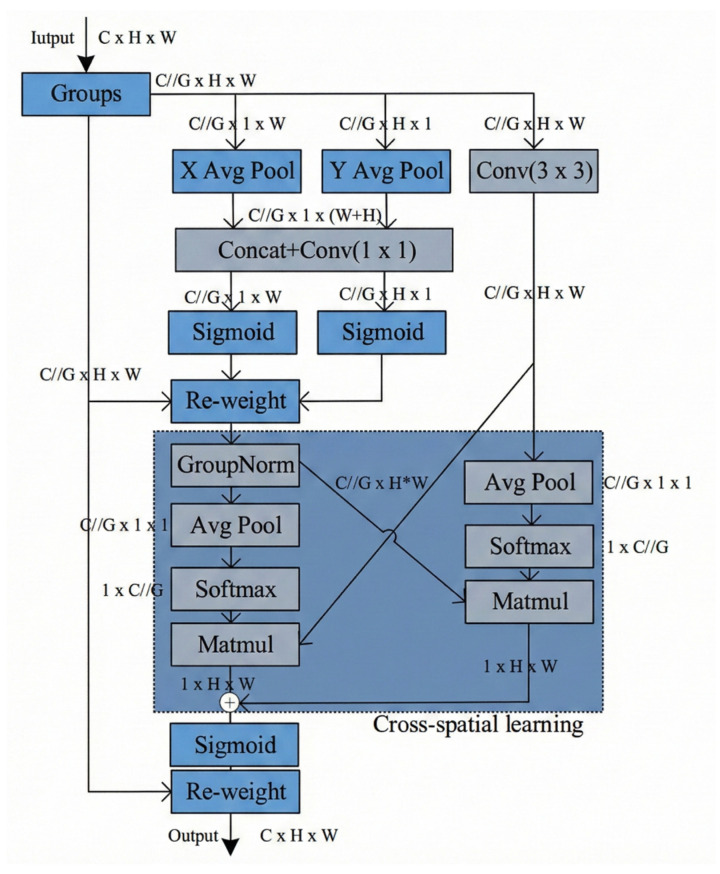
C3EMA network.

Given the feature distribution with multi-scale characteristics and complex local textures on the surface of composite materials, traditional convolution struggles to characterize both long-range dependencies and global context simultaneously due to its limited receptive field. To address such issues, we introduce the Efficient Multi-Scale Attention (EMA) mechanism, as illustrated in Figure 5. The EMA module adopts a multi-branch parallel structure combined with a cross-spatial learning and fusion mechanism as a whole, and each branch collaboratively functions to realize its core capabilities.

Among them, the local-scale spatial attention branch is mainly responsible for capturing local features such as fine textures, edges and small targets in the feature map. This branch extracts local spatial responses by adopting structures such as 3 × 3 depthwise separable convolution and generates a spatial weight map to perform weighted enhancement on local features. This design not only ensures computational efficiency but also effectively preserves high-resolution local details. Complementary to the local-scale branch, the global-scale spatial attention branch focuses on capturing large-range contextual information, long-range dependency relationships and global structural features. Abandoning the standard self-attention structure with extremely high computational complexity, this branch constructs a large receptive field through a lightweight design combining pointwise convolution with global average or max pooling and generates a global spatial weight map. In this way, it enhances the feature representation of important global regions such as the target main body and large structures in the feature map, which effectively mitigates the problems of over-refined local description and insufficient global perception in traditional lightweight attention mechanisms, enabling the model to better understand the overall scene layout.

In addition to the two spatial-scale branches, the EMA module also incorporates a channel attention branch, which adopts a more lightweight squeeze-excitation structure similar to that of the Squeeze-and-Excitation (SE) module. This branch generates channel weight maps via global pooling, 1 × 1 convolution and activation functions, achieving adaptive enhancement of important feature channels and suppression of useless ones. It forms a synergistic effect with the spatial attention branches, thus constructing a spatial-channel joint attention mechanism, which compensates for the limitations of single spatial or channel attention mechanisms. This design further enhances the discriminative ability of features without significantly increasing the overall computational overhead of the model.

- CARAFE

**Figure 6:**
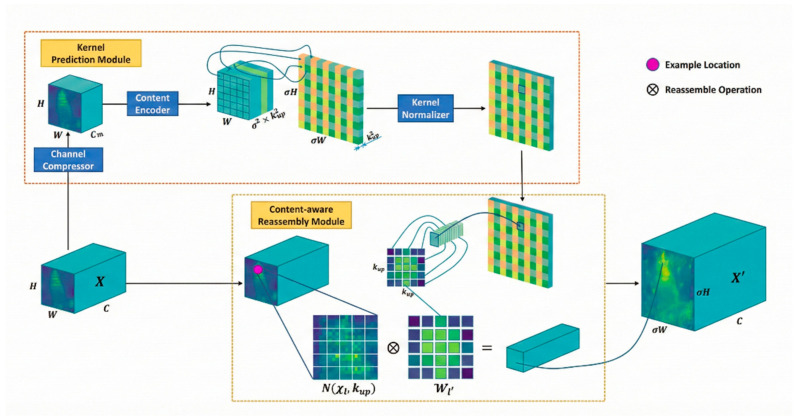
CARAFE network.

Images of composite materials are often characterized by complex scenes, strong interference and significant scale variations of targets. Moreover, the targets exhibit remarkable scale disparities, with micro-scale subtle damages, fiber distortions and centimeter-scale overall structural regions coexisting simultaneously. Traditional fixed-kernel upsampling methods such as bilinear interpolation tend to over-smooth in high-interference regions and lose the edge and texture details of weak targets, while transposed convolution is prone to generating checkerboard artifacts in regions with complex textures, which further exacerbates noise interference. In contrast, CARAFE dynamically generates upsampling kernels adapted to local regions through content awareness; it can adaptively suppress irrelevant background responses in noisy and high-interference regions and enhance feature weights in target edge and fine texture regions. Thus, it preserves real and valid structural information while improving resolution and effectively suppresses clutter and artifacts. Therefore, this paper replaces the original upsampling module with CARAFE, whose network structure is illustrated in Figure 6.

Unlike methods with fixed interpolation kernels, CARAFE can dynamically generate upsampling kernels based on the local content of input features. It maintains a large receptive field while keeping extremely low computational and parameter overhead, thus achieving efficient and high-precision feature reassembly.

The CARAFE module is composed of two core components: the upsampling kernel prediction module and the feature reassembly module. The former is responsible for generating spatially variable upsampling kernels from input features, while the latter performs weighted reassembly on the original feature maps using the predicted kernels, thereby obtaining high-resolution feature maps.

In the upsampling kernel prediction module, channel compression is first applied to the input feature maps to reduce the subsequent computational load. The compressed feature map can be expressed as:

*ϕ*(*X*) = Conv_1×1_(*X*), (1)

where X ∈ R^C×H×W^ denotes the input feature, and Conv_1×1_ stands for the 1 × 1 convolution.

Subsequently, the compressed features are used to generate an upsampling kernel map of size k × k with an upsampling scale of α, which yields the reassembly weights corresponding to each output position. This process can be expressed as:

*W* = *σ*(Conv*_k×k_*(*ϕ*(*X*))), (2)

where Conv_k×k_ denotes the convolutional layer encoding local context, with k typically set to 3 or 5 to balance the receptive field and computational load; σ represents the Sigmoid activation function, which normalizes the kernel weights to the range of 0 to 1. The finally obtained kernel map is $W\;\in \;R^{\alpha^{2}k^{2}\times H\times W}$, where each spatial position corresponds to α^2^ k × k upsampling kernels.

In the feature reassembly stage, CARAFE performs weighted reassembly on the original low-resolution features using the generated dynamic kernels, yielding high-resolution output features. For each position (i,j) in the target upsampled feature map, its feature value is obtained by the weighted summation of the features in the corresponding neighborhood of the original feature map and the dynamic kernel, and the calculation method is expressed as:

*Y*_*i*,*j*_ = ∑_*dx*,*dy*_*W*_*i*,*j*_(*dx*,*dy*)⋅*X*_⌊*i*/*α*⌋+*dx*,⌊*j*/*α*⌋+*dy*_, (3)

where Y denotes the output high-resolution feature map, ⌊⋅⌋ is the floor operation for locating the reference position of the original feature map, dx and dy are the offsets within the kernel window, and the weighted summation is completed by traversing the entire k × k kernel space.

Faced with the characteristic of multi-scale coexistence of targets in composite materials, CARAFE relies on the larger receptive field brought by convolutional kernels to naturally aggregate multi-scale contextual information during the upsampling process. It can not only accurately reconstruct the fine-grained structures of small-scale defects but also maintain the semantic consistency of large-scale structural regions, thus avoiding the problems of disappearance of small targets and blurred edges of large targets of multi-scale objects after upsampling. Meanwhile, its lightweight structure will not introduce significant computational burden with the increase in image complexity and can be stably embedded into network architectures for composite material detection and segmentation. This significantly improves the feature reconstruction quality and final recognition and localization accuracy under the conditions of complex interference and drastic scale variations.

#### 3.1.3. Image-to-Point Segmentation

After obtaining the accurate segmentation masks of the composite material surface in visible light images via the Yolo-Mask network, we describe the specific method for converting these masks into the 3D point cloud of the material surface. The core of this process lies in leveraging the calibrated pixel-level correspondence between visible light images and depth maps in the structured light camera system, as well as the rigid transformation formula between depth maps and 3D point clouds. The specific calculation process is as follows:

(4) $$\begin{array}{c} z=d/s\\ x=(u-c_{x})\cdot z/f_{x}\\ y=(v-c_{y})\cdot z/f_{y} \end{array},$$

where f_x_ and f_y_ denote the focal lengths of the camera along the x and y axes, c_x_ and c_y_ are the coordinates of the camera’s aperture center, and s is the scaling factor of the depth map.

During this step, we first treat the binary segmentation masks output by the network as the Region of Interest (ROI) and directly map them onto the synchronously acquired depth maps. This operation is equivalent to masking the depth maps with the masks to retain only the valid depth values of the composite material surface regions, thus yielding the segmented depth maps.

Subsequently, based on the camera’s intrinsic parameters, the 2D coordinates and corresponding depth values of each pixel in the segmented depth maps are converted into point cloud coordinates in 3D space via coordinate transformation formulas. After this conversion, the segmented 3D point cloud data of the composite material surface are obtained. These data as the precise input for subsequent robotic arm scanning path planning, effectively eliminating interference from the background and irrelevant objects, and thus ensuring the targeting and safety of the scanning operation.

### 3.2. Three-Dimensional Vision-Guided Robotic Arm Ultrasonic Automatic Scanning Method

During the scanning process, first, the robotic arm carrying the binocular camera moves to the preset imaging pose, captures the visible light images, depth maps and 3D point clouds of the workpiece to be inspected, and acquires the point cloud data of the test piece.

Subsequently, the point cloud of the workpiece to be inspected undergoes a series of processing operations via the point cloud data processing module. Based on the calibration parameter results from camera calibration, hand-eye calibration, and tool calibration, as well as the ultrasonic testing trajectory points selected after point cloud processing, the six-dimensional coordinates of the trajectory points for robotic arm movement are calculated.

Then, the robotic arm is controlled to move along the trajectory points, thereby performing the ultrasonic testing task.

#### 3.2.1. Surface Point Cloud-Based Trajectory Point Selection Method

- Voxel Grid-Based Point Cloud Downsampling

The large volume of point cloud data acquired by the structured light camera renders subsequent processing time-consuming. Downsampling is an effective method for reducing data volume and cutting down computational load. Point cloud downsampling is intended to convert operations on the entire point cloud to the key points obtained via downsampling, thereby achieving the goal of reducing computational load. The core of voxel grid downsampling lies in dividing the 3D space into uniform voxels and generating a representative point within each non-empty voxel to realize data compression [35]. Given the original point cloud P = {p_1_,p_2_,…,p_n_}, where each point p_i_ = (x_i_,y_i_,z_i_) represents 3D spatial coordinates, their bounding range is defined as:

(5) $$\left\{\begin{array}{c} x_{\min}=min\left(x_{i}\right),x_{\max}=max\left(x_{i}\right)\\ y_{\min}=min\left(y_{i}\right),y_{\max}=max\left(y_{i}\right)\\ z_{\min}=min\left(z_{i}\right),z_{\max}=max\left(z_{i}\right) \end{array}\right.$$

Subsequently, the 3D space is divided into a regular grid according to the preset voxel size v. For any point p_i_, the index of its corresponding voxel can be determined via a mapping function. After completing the spatial division, for the point set contained in each non-empty voxel, the representative point is calculated using the centroid, which is expressed as:

(6) $${\hat{p}}_{j}=\frac{1}{\left|P_{j}\right|}\underset{p\in P_{j}}{\sum }\;p,$$

This representative point not only preserves the geometric center characteristics of the local region but also effectively reduces data redundancy. The voxel grid downsampling method effectively reduces the data volume through spatial aggregation while preserving the macroscopic geometric structure of the point cloud, making it suitable for real-time processing and the preliminary simplification of large-scale point cloud data.

- Local Surface Fitting-Based Point Cloud Normal Vector Calculation

The ultrasonic probe must be perpendicular to the workpiece surface during ultrasonic scanning. The normal vector information of the workpiece surface point cloud directly acquired by the 3D camera is not fully accurate and exhibits a certain deviation from the ideal value, so it is necessary to recalculate the normal vector information of the workpiece surface point cloud.

The local neighborhood of any point in the point cloud can be effectively fitted by a tangent plane, provided that the surface is sufficiently smooth [36]. The specific implementation process is as follows: For any scanning point p in the workpiece point cloud, first, its K nearest neighbors are obtained via nearest neighbor search to form a local point set; then, based on this point set, an optimal local plane P is constructed using the least squares fitting method, whose mathematical expression is:

(7) $$P(\vec{n},d)=\underset{\left(\vec{n},d\right)}{\mathrm{argmin}}\sum_{i=1}^{k}{\left(\vec{n}\cdot p_{i}-d\right)}^{2},$$

where $\vec{n}$ denotes the normal vector of plane **P**, and d is the distance from plane **P** to the coordinate origin.

When the local plane formed by the K nearest neighbors and the scanning point **P** is sufficiently small, it can be considered that the normal vector of this plane **P** is fitted by the K nearest neighbors, which is also the normal vector of the current scanning point **P**.

By performing eigenvalue decomposition on the covariance matrix **M**, the eigenvalues of **M** are obtained. The eigenvector corresponding to the smallest eigenvalue of **M** is the normal vector of plane **P**. Thus, the normal vector information of the scanning point p can be obtained.

(8) $$M=\frac{1}{k}\sum_{i=1}^{k}\left(p_{i}-p_{0}\right){\left(p_{i}-p_{0}\right)}^{T},$$

$p_{0}$ is the centroid.

- Principal Component Analysis (PCA)-Based Point Cloud Region Segmentation

Since the area of the workpiece to be scanned is generally large while the scanning range of the ultrasonic probe is limited, it is necessary to perform region segmentation on the workpiece point cloud. The purpose is to divide the workpiece point cloud into elongated regions that allow unidirectional scanning by the ultrasonic probe.

Point cloud region segmentation aims to decompose the complete 3D point cloud dataset into multiple sub-regions with clear directionality according to specific geometric features. We will use Principal Component Analysis (PCA) to determine the principal directions of the point cloud, and based on this, realize directional region segmentation of the point cloud.

In 3D point cloud processing, PCA [37] can effectively identify the main distribution directions of point cloud data, providing a geometric basis for subsequent region segmentation. Let the point cloud dataset contain *n* 3D points, denoted as $P=p_{i}={\left(x_{i},y_{i},z_{i}\right)}^{T}|i=1,2,\ldots ,n$, where each point also contains normal vector information $n_{i}={\left(n_{x_{i}},n_{y_{i}},n_{z_{i}}\right)}^{T}$. The centroid coordinates of all points are:

(9) $$\bar{p}=\frac{1}{n}\overset{n}{\underset{i=1}{\sum }}\;p_{i}={\left(\bar{x},\bar{y},\bar{z}\right)}^{T},$$

By subtracting the centroid coordinates from the coordinates of all points, we obtain the centered point cloud data $p_{i}^{\prime }$ = $p_{i}$ − $\bar{p}$. The centering operation eliminates the translation component of the point cloud data, ensuring that subsequent covariance calculations can truly reflect the distribution characteristics of the data. Then, a covariance matrix **C** is constructed, which describes the dispersion and correlation of the point cloud data along different coordinate axes and can be defined as:

(10) $$C=\frac{1}{n}\overset{n}{\underset{i=1}{\sum }}\;p_{i}^{\prime }{\left(p_{i}^{\prime }\right)}^{T}=\left[\begin{array}{ccc} \sigma_{xx} & \sigma_{xy} & \sigma_{xz}\\ \sigma_{yx} & \sigma_{yy} & \sigma_{yz}\\ \sigma_{zx} & \sigma_{zy} & \sigma_{zz} \end{array}\right],$$

where σ_jk_ denotes the covariance between the j-th and k-th coordinate dimensions.

Singular value decomposition (SVD) is performed on the centered point cloud data matrix $X={\left[p_{1}^{\prime },p_{2}^{\prime },\ldots ,p_{n}^{\prime }\right]}^{T}$:

(11) $$X=U\Sigma V^{T},$$

where the column vectors of **V** are the eigenvectors of the covariance matrix, and the squares of the corresponding singular values are proportional to the eigenvalues. The eigenvector **V_1_** corresponding to the largest eigenvalue represents the principal direction of the point cloud data. The eigenvector **V_2_** corresponding to the second-largest eigenvalue represents the secondary direction of the point cloud data. The eigenvector **V_3_** corresponding to the smallest eigenvalue is usually perpendicular to the main distribution plane of the point cloud.

After determining the principal and secondary directions, directional region segmentation can be performed. Let the principal direction vector be **d_main_** and the secondary direction vector be **d_sec_**. Each point $p_{i}$ is projected onto the principal direction, and the projection value is $t_{i}=p_{i}\cdot d_{\mathrm{main}}$. The range of all projection values [$t_{\min}$, $t_{\max}$] is calculated, where $t_{\min}={min}_{i}\cdot \;t_{i}$. $t_{\max}=max_{i}\cdot t_{i}$.

We uniformly place m + 1 segmentation positions along the principal direction to divide the point cloud into m elongated regions along the principal direction:

(12) $$t_{j}=t_{\min}+\frac{j\left(t_{\max}-t_{\min}\right)}{m},j=0,1,\ldots ,m,$$

Among them, the j-th region contains all points satisfying $t_{j}\leq t_{i}<t_{j+1}$. This segmentation method ensures that each region has a similar length along the principal direction, forming elongated strip-like structures.

To further refine the geometric shape of the regions, constraints can be imposed along the secondary direction. For the point set P_j_ within the j-th region, its projection range is calculated along the secondary direction:

(13) $$s_{j,\min}=\underset{p_{i}\in P_{j}}{min}\left(p_{i}\cdot d_{\sec}\right),\;s_{j,\max}=\underset{p_{i}\in P_{j}}{max}\left(p_{i}\cdot d_{\sec}\right),$$

By setting a width threshold w along the secondary direction, we can filter out the points that satisfy $s_{j,\min}\leq p_{i}\cdot d_{\sec}\leq s_{j,\min}+w$, obtaining elongated rectangular regions with controllable width. This bidirectional-constraint segmentation strategy can better preserve the integrity of local geometric features when dealing with complex curved surface structures.

When saving the segmentation results, in addition to the 3D coordinates (x,y,z) of the points, the normal vector information (n_x_,n_y_,n_z_) of the original point cloud is also retained, so the complete regional data format is a six-dimensional vector (x,y,z,n_x_,n_y_,n_z_).

- Equal Interval Sampling-Based Center Trajectory Point Extraction Method

After completing region segmentation of the workpiece point cloud, it is necessary to extract the trajectory points for the ultrasonic probe to perform scanning motions from each divided elongated region. To ensure that the ultrasonic probe covers the entire width of the elongated region, the scanning trajectory points should lie on the centerline of each elongated region. Additionally, to ensure that the ultrasonic probe remains in full contact with the workpiece surface throughout the scanning process, the spacing between scanning trajectory points should be appropriately adjusted according to the curvature of the workpiece surface.

The centerline trajectory point extraction method we propose is based on the geometric foundation obtained via PCA, with a focus on constructing an accurate equal interval sampling strategy along the determined principal direction. As previously described, the principal direction vector **v_1_** and centroid $\bar{p}$ of the point cloud have been obtained using the PCA method. We project the point cloud onto the principal direction and calculate the projection scalar value of each point **p_i_**:

(14) $$s_{i}={\left(p_{i}-\bar{p}\right)}^{⊤}v_{1},$$

From this, we further determine the boundary values of the projection range: $s_{\min}=min_{i}s_{i}$ and $s_{\max}=max_{i}s_{i}$. Based on these boundary values, M equal interval sampling parameters are generated along the principal direction:

(15) $$t_{j}=s_{\min}+\frac{j}{M-1}\left(s_{\max}-s_{\min}\right),$$

where j = 0, 1, …, M − 1, the corresponding theoretical spatial point coordinates are $q_{j}=\bar{p}+t_{j}v_{1}$. To ensure that the extracted points originate from the original point cloud and retain their intrinsic properties, a nearest neighbor search strategy is used to locate the actual trajectory points. For quality control, a distance threshold δ is introduced: if $min_{p_{i}\in P}|p_{i}-q_{j}{|}_{2}$, a warning is issued to indicate a potential fitting deviation. The final trajectory point set not only contains coordinate information but also retains the original normal vector information, forming complete six-dimensional data trajectory points.

#### 3.2.2. Three-Dimensional Point Cloud-Based Robotic Arm Motion Trajectory Calculation Method

Robotic arm pose estimation under visual guidance is the core link for achieving automated ultrasonic scanning, the goal of which is to determine the accurate pose of the robotic arm end effector along the scanning trajectory based on 3D visual information. This paper realizes 3D point cloud-based robotic arm motion trajectory generation and pose calculation through the following steps:

A binocular structured light camera is used for 3D scanning of the workpiece surface to obtain high-precision surface point cloud data. Subsequently, the material surface point cloud is extracted from the point cloud via the YOLO-Mask module. Next, the material surface point cloud is preprocessed, including point cloud filtering and denoising, normal vector estimation, region segmentation, and scanning trajectory planning, to finally extract the 3D coordinates and normal vector directions of the points to be scanned in the camera coordinate system.

After obtaining the trajectory point poses in the camera coordinate system, the poses of the target points in the robotic arm base coordinate system are calculated via coordinate chain transformation, by combining the hand-eye calibration relationship, tool calibration parameters, and the joint states of the robotic arm wrist when the point cloud was collected. This pose is the end-effector target pose used to drive the robotic arm for ultrasonic scanning.

Finally, based on this pose, fully automated ultrasonic scanning of the aircraft composite laminated structure by the robotic arm is realized, and the probe pose data and ultrasonic signal data during scanning are saved in real time, providing basic coordinate and signal data for subsequent 3D ultrasonic imaging.

### 3.3. Method for Fusing Robotic Arm Pose and Ultrasonic Data

The specific implementation process of the Method for Fusing Robotic Arm Pose and Ultrasonic Data starts with a pre-planned robotic arm scanning path, where the end effector holds an ultrasonic probe to perform automated full-area scanning on the surface of the composite material component to be inspected.

During this process, the system synchronously records two core data types:

1. The six-dimensional pose data of the probe in the global coordinate system, fed back by the high-precision encoder of the robotic arm;
2. The full-sequence ultrasonic B-scan data containing internal material information, collected by the phased array ultrasonic probe.

The ultrasonic B-scan data undergo LVAE (Latent Variable Autoencoder)-based signal classification. Specifically, the LVAE network adopts an encoder–decoder structure: the encoder consists of four fully connected layers with 256, 128, 64, and 32 neurons, respectively, while the decoder mirrors this structure with 32, 64, 128, and 256 neurons; the latent space dimension is set to 16, with ReLU as the activation function and a composite loss function combining mean squared error (MSE) for reconstruction and cross-entropy for classification. During training, the batch size is 32, the number of epochs is 100, the learning rate is 1 × 10^−4^, and the Adam optimizer is adopted. For signal classification, the latent space features are mapped to three signal categories (normal, damage, noise), and a fixed threshold is set based on the reconstruction error distribution to distinguish damage signals from others.

Subsequently, segmented filtering and depth compensation are performed on the classified signals. For segmented filtering, wavelet threshold filtering is adopted: the Daubechies 4 (db4) wavelet basis function is selected, with a decomposition scale of 5 levels, and adaptive threshold values are set for different signal segments to suppress noise while preserving damage echo features. For depth compensation, a mathematical model is established: The sound velocity correction model accounts for the variation in ultrasonic velocity with composite material thickness, the time delay compensation formula corrects the echo arrival time based on propagation distance, and the probe coupling loss is compensated using an exponential function related to depth, ensuring the accuracy of echo positioning in the depth direction [38,39].

Then, each processed ultrasonic B-scan datum is accurately transformed from the probe coordinate system to the global coordinate system (referenced to the composite laminated structure) according to its corresponding spatial pose matrix.

Next, via the voxel backprojection algorithm, the damage echo energy reflected in all ultrasonic data is filled and accumulated one by one into a 3D voxel space model. The core parameters of voxel backprojection are set as follows: the voxel resolution is 0.05 mm × 0.05 mm × 0.05 mm in the X/Y/Z directions; trilinear interpolation is used to map echo points to voxel coordinates; the echo energy accumulation rule follows the linear superposition of normalized echo amplitude; and an exponential attenuation model based on composite material acoustic impedance is applied to compensate for ultrasonic energy loss with propagation depth. In terms of computational implementation, the voxel space is constructed to cover the entire scanned area of the composite component, the probe pose is converted to voxel coordinates through homogeneous transformation, and a GPU-based parallel computing strategy is adopted to accelerate the processing of large-scale voxel data.

Finally, a 3D ultrasonic atlas is generated, which clearly displays the three-dimensional morphology, spatial distribution, and distance from the surface layer of internal damage in the composite material, and intuitively presents the area of delamination regions, the depth of debonding, or the three-dimensional contour of impact damage.

Comparative experiments using two classic network models are conducted to verify the superiority of the proposed ultrasonic signal classification network. The comparative models include the Autoencoder (AE), an unsupervised model that learns compressed data representations via an encoder–decoder structure, and the Variational Autoencoder (VAE), a generative model that introduces probability distributions on the basis of AE to generate new data. The detailed results are presented in Table 1.

Table 1 shows the comparison results between the proposed LVAE and other network models. As can be seen from the table, the LVAE model significantly outperforms both AE and VAE in terms of the AUC metric under different gain conditions (10 dB, 15 dB, and 20 dB). The average AUC value of LVAE reaches 0.980, which is 0.131 higher than that of AE and 0.083 higher than that of VAE, indicating that the proposed LVAE can classify signals more accurately. Meanwhile, in terms of inference efficiency, LVAE can classify 163 signals per second, meeting the engineering requirements of real-time detection. Therefore, the proposed LVAE model not only surpasses the traditional AE and VAE in classification accuracy, but also exhibits favorable computational efficiency, making it more suitable for the fast and reliable ultrasonic detection of aircraft composite laminate structures.

## 4. Experiment and Discussion

### 4.1. Experimental Setup and Evaluation Metrics

The experimental environment consisted of a Windows 10 operating system, an NVIDIA RTX 3090 (24 GB) graphics card, Python version 3.12, PyTorch version 2.6, and CUDA version 12.1.

To comprehensively evaluate the performance of the composite surface instance segmentation network, the following parameter settings were adopted for the model training process: the input image size was set to 1200 × 800, the batch size was 8, and the entire training process was conducted for 300 epochs. Stochastic Gradient Descent (SGD) was used for training, with a weight decay of 0.0005 and a momentum of 0.937. The training was divided into two phases: the learning rate was set to 0.01 for the first 100 epochs and adjusted to 0.001 for the subsequent 200 epochs.

Precision (P), Recall (R), and Average Precision (AP) are the most common evaluation metrics in deep learning models. Intersection over Union (IoU) refers to the ratio of the intersection to the union between the bounding box and the ground truth box. Meanwhile, mean Average Precision (mAP) and detection speed (frames per second, fps) are typically used as evaluation metrics in composite surface instance segmentation. mAP is further divided into Bbox-mAP and Segm-mAP, representing the mAP for detection and segmentation, respectively. Based on the above parameters, the metrics for evaluating the performance of the model proposed in this paper are obtained as follows:

(16) $$\begin{array}{l} Precision=\frac{TP}{TP+FP}\\ Recall=\frac{TP}{TP+FN}\\ AP=\int_{0}^{1}Precision(Recall)d(Recall)\\ mAP=\frac{1}{N}\sum_{i=1}^{N}AP_{i} \end{array}$$

### 4.2. Experiment of Composite Laminated Structure Instance Segmentation

#### 4.2.1. Comparative Experiment

We conducted comparative experiments on the regional segmentation of composite laminated structures and image-to-point cloud segmentation experiments and carried out a detailed analysis and discussion on the performance of the YOLO-Mask network proposed above. Comparative experiments were performed on the self-built dataset with typical segmentation models in recent years, which are YOLO11 [40], Mask R-CNN [41] and DynaMask [42], respectively. The results of the comparative experiments are presented in Table 1.

Table 1 presents the comparison results of YOLO-Mask with other benchmark models. In terms of core accuracy metrics, YOLO-Mask ranks first with a Bbox-mAP of 98.7% and a Segm-mAP of 97.4%. Even in comparison with DynaMask, the state-of-the-art model with excellent performance, the Segm-mAP of YOLO-Mask is 1.6% higher. In terms of speed, YOLO-Mask is the fastest among networks with segmentation capabilities, reaching 11.7 fps. In conclusion, the network structure proposed in this paper can effectively accomplish the segmentation task for composite laminated structure regions.

**Figure 7:**
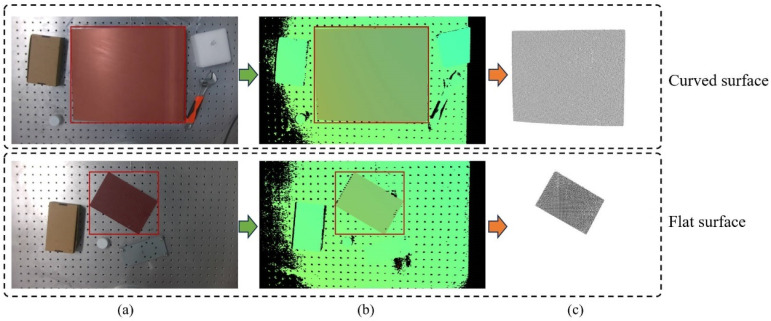
The result of image-to-point cloud: (**a**) Visible light image segmentation, (**b**) depth map segmentation, (**c**) surface point cloud.

Figure 7 shows the segmentation results of image-to-point cloud conversion. As can be seen from the figure, after the YOLO-Mask network segments the composite material regions in visible light images, the segmentation of the depth map is realized by mapping the segmented regions to the depth map; finally, the segmented material surface point cloud is obtained via conversion based on the segmented depth map.

#### 4.2.2. Ablation Experiment

This study specifically analyzed the impact of the C3EMA module and CARAFE module on model instance segmentation capability through ablation experiments. See Table 2 for details.

Table 3 presents the results of the ablation experiments. Optimization with C3EMA alone increased the Segm-mAP value by 2.4% (from 92.2% to 94.6%), with the detection speed decreasing by 3.0 fps. Optimization with CARAFE alone raised the Segm-mAP value by 3.7% (from 92.2% to 95.9%), and the detection speed dropped by 5.3 fps. The combined use of both modules increased the Segm-mAP value by 5.2% (from 92.2% to 97.4%), with a 6.9 fps decrease in detection speed. These results indicate that both improvement methods can effectively enhance the detection performance of the model.

The underlying reasons for the results in Table 3 lie in the fact that the optimization modules of C3EMA and CARAFE are complementary, lightweight and synergistic: their separate or combined application all leads to an increase in Segm-mAP, while the fps decreases gradually with the rise in computational complexity. C3EMA integrates the EMA module to optimize feature extraction, which enhances defect features and suppresses interference via multi-scale attention and only brings a slight increase in computational complexity, thus resulting in an increase in mAP and a moderate decrease in fps. CARAFE optimizes upsampling and mask generation, and its dynamic kernel can accurately reconstruct defect contours with a computational complexity slightly higher than that of C3EMA, leading to a more pronounced drop in fps.

To rigorously assess the deployment readiness of the robotic inspection system, we conducted a comprehensive end-to-end cycle-time analysis, decomposing the full workflow into six sequential stages that directly determine system throughput:

(1) Visible light/depth image acquisition
(2) Point cloud generation and YOLO-Mask-based segmentation
(3) Point cloud processing and robotic scanning trajectory planning
(4) Robotic arm motion and ultrasonic data acquisition (with sensor synchronization)
(5) LVAE-based ultrasonic signal processing and data fusion
(6) Voxel backprojection-based 3D reconstruction and defect visualization

We measured the time consumption of each stage on composite test pieces of three representative sizes (100 × 100 mm, 300 × 300 mm, 500 × 500 mm) and calculated the total cycle time (from image capture to final 3D defect visualization) and effective detection efficiency (mm^2^/s) for each configuration. The results (summarized in Table 4) demonstrate that the system maintains stable throughput across different component scales, with effective detection efficiency meeting the requirements of aerospace engineering inspection.

Furthermore, we evaluated the system’s throughput scaling behavior with respect to key parameters:

Scan area: As the scan area increases from 100 × 100 mm to 500 × 500 mm, the total cycle time increases linearly, while the effective detection efficiency remains within 85–92 mm^2^/s, confirming consistent throughput performance.

Sampling density and voxel resolution: We tested three levels of sampling density (low/medium/high) and voxel resolutions (0.1 mm/0.05 mm/0.02 mm). The results show that higher sampling density and finer voxel resolution improve 3D reconstruction accuracy but increase the time consumption of ultrasonic data acquisition and 3D reconstruction. The parameter combinations adopted in this study (medium sampling density, 0.05 mm voxel resolution) strike a balanced trade-off between reconstruction precision and throughput, which is well-aligned with typical aerospace NDT inspection requirements.

### 4.3. Experiment of 3D Vision-Guided Ultrasonic Scanning

**Figure 8:**
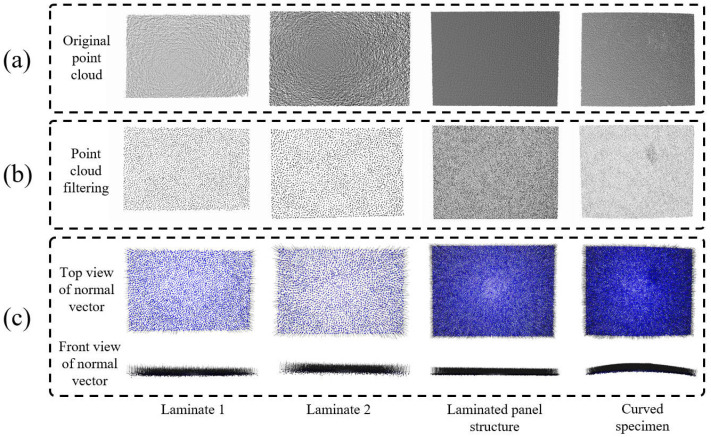
Experiment results of sampling and normal vector calculation in 3D point cloud: (**a**) Oringinal point cloud, (**b**) Point cloud filtering, (**c**) Normal vector.

**Figure 9:**
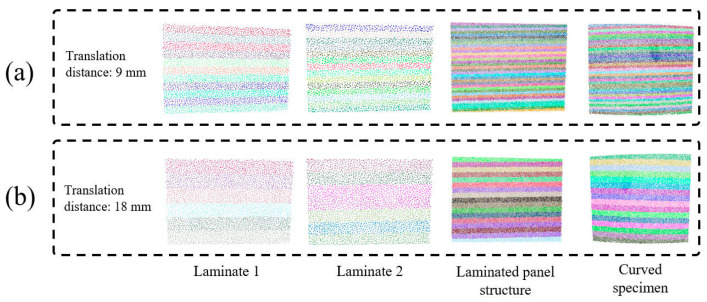
Experimental results of 3D point cloud region partitioning: (**a**) Translation distance: 9 mm, (**b**) Translation distance: 18 mm.

**Figure 10:**
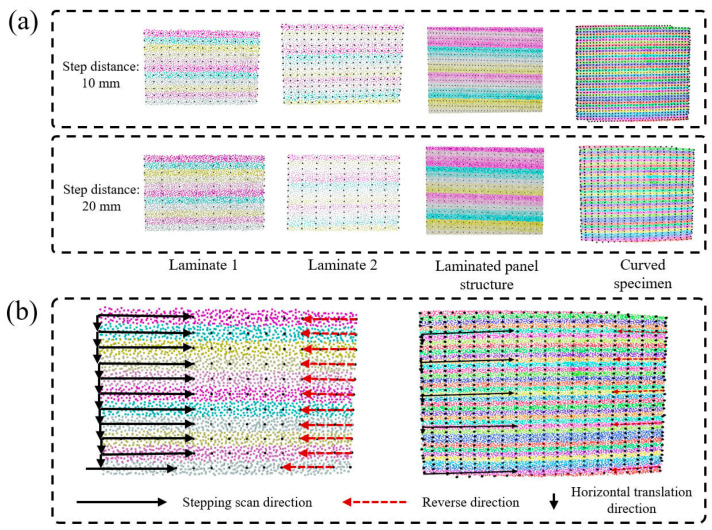
Experimental results of 3D point cloud region center trajectory point selection and schematic diagram of scanning direction: (**a**) Step distance, (**b**) Scanning direction.

To verify the effectiveness and practicability of the adopted 3D point cloud data processing method, comprehensive experimental tests were conducted on four workpieces with different geometric complexities in this section, and the experimental results are shown in Figure 8, Figure 9 and Figure 10. The entire experimental process covered the complete technical route from the acquisition of original point cloud data to the generation of the final scanning trajectory, which fully verified the feasibility and accuracy of all the technical methods involved.

Figure 8 presents the experimental results of 3D point cloud downsampling and normal vector calculation. The experimental data shown in Figure 8b indicate that the voxel grid downsampling method significantly reduces the point cloud data density while maintaining the integrity of the workpiece’s geometric features. It can be observed from the experimental results that the downsampled point cloud data still maintain good shape fidelity at the key geometric features of the workpiece, with its edge contours and surface details effectively preserved. Meanwhile, the normal vector calculation method based on local surface fitting has successfully generated accurate normal vector information for each downsampled point, and these normal vectors exhibit excellent directional consistency and spatial continuity. From the experimental results in Figure 8c, it can be seen that even in the regions of the workpiece surface with large curvature variations, the calculated normal vectors can still accurately reflect the geometric characteristics of the local surface.

Figure 9 presents the experimental results of 3D point cloud region segmentation, which demonstrate the excellent performance of PCA in processing workpieces with different complexities. As can be seen from the figure, for the four test workpieces with distinct geometric features, PCA can accurately identify the principal directions of the point clouds and realize effective directional region segmentation accordingly. The selection of experimental parameters exerts a significant impact on the segmentation results, among which the setting of the translation distance parameter directly determines the width and quantity distribution of elongated regions. As shown in Figure 9a, when the translation distance is set to a small value, the elongated regions become narrower; this improves scanning fineness but simultaneously increases the complexity of the scanning path and the total scanning time. In contrast, as shown in Figure 9b, a large translation distance results in relatively wider elongated regions with more abundant point cloud data contained in each region, yet it may lead to an excessively wide single scanning coverage and thus compromise detection accuracy.

It can also be found from the experimental results that the geometric complexity of the workpiece surface has a crucial influence on the effect of region segmentation. For workpieces with relatively flat surfaces and simple geometric features, PCA can clearly identify distinct principal and secondary directions, yielding clear segmentation boundaries and good geometric consistency among all regions. However, for workpieces with complex curved surface features, although PCA can still effectively capture the overall geometric trend, a certain degree of irregularity may occur at the boundaries of local regions. This phenomenon is reflected in the experimental results as slightly irregular geometric shapes of some elongated regions, while the regions still retain good directional characteristics on the whole (as shown in Figure 9). Therefore, for workpieces with different geometric complexities, there exists an optimal parameter configuration for region segmentation, which requires finding a proper balance between accuracy and efficiency.

Figure 10 presents the experimental results of center trajectory point selection for 3D point cloud regions and the schematic diagram of the scanning direction. As can be seen from the figure, the proposed centerline extraction method is capable of generating high-quality scanning trajectories under different parameter configurations. The experimental results in Figure 10a show that the density parameter of trajectory points exerts a crucial influence on the final trajectory quality. When the trajectory point density is set to a high value, the generated scanning path becomes smoother and finer, which can better adapt to the curvature variations of the workpiece surface and ensure that the ultrasonic probe maintains good contact with the workpiece surface throughout the scanning process. However, an excessively high trajectory point density will also lead to a reduction in scanning efficiency and an increase in computational complexity. In addition, the setting of the distance threshold parameter δ plays an important role in the precision control of trajectory point extraction. It can be observed from the experimental results that an appropriate distance threshold can effectively filter out abnormal points that deviate too far from the centerline, ensuring that the extracted trajectory points truly reflect the central positions of the elongated regions.

The schematic diagram of the scanning direction is also presented in Figure 10b. For any elongated region, the scanning step is first performed along the principal direction of the region; upon reaching the last trajectory point of the region, a retraction motion is executed to return to the starting point of the region, followed by a lateral translational movement to the starting point of the next region. After arriving at the starting point of the next region, the scanning step for the next region is carried out. This process is repeated until the scanning of all segmented regions is completed. This scanning strategy can effectively ensure the continuity and integrity of ultrasonic testing data. This back-and-forth scanning mode avoids the abrupt direction change problem that may be caused by the traditional serpentine scanning, reduces the frequency of probe pose adjustment, and improves the stability of scanning.

A comprehensive analysis of the experimental results of the four workpieces reveals that the proposed 3D point cloud data processing method exhibits excellent adaptability and robustness. After undergoing the complete point cloud processing workflow, workpieces with different geometric complexities can all generate high-quality scanning trajectories, which verify the universality of the method. The experimental results also indicate that through rational parameter configuration and optimization, this method can realize efficient automated ultrasonic scanning path planning on the premise of ensuring detection accuracy.

### 4.4. Experiment on Fusion of Manipulator Pose and Ultrasound Data

To verify the effectiveness and generalization ability of the proposed robotic arm pose and ultrasonic data fusion method in practical applications, we conducted a systematic analysis and discussion on the inspection and imaging results of two types of typical test pieces, including damage-simulated test pieces of composite laminated structures and damage-simulated test pieces of composite laminated plates.

#### 4.4.1. Damage Simulation Specimen of Composite Laminated Structure

**Figure 11:**
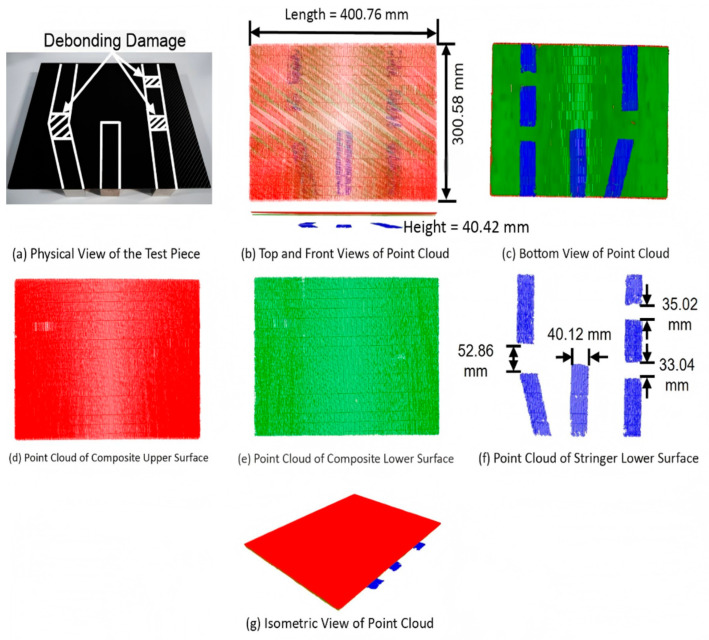
Intelligent ultrasonic testing results of damage simulation specimen of composite laminated structure.

We conducted fusion detection experiments on the damage-simulated test piece of the composite laminated structure, and the final 3D ultrasonic inspection result map was obtained after processing via the method proposed in this paper. Figure 11 presents the intelligent ultrasonic inspection results of the damage-simulated test piece of the composite laminated structure. Among them, Figure 11a is the physical drawing of the test piece; as can be seen from the figure, the test piece consists of a composite flat plate and three aluminum alloy stringers with different shapes, with three precast debonding damages. Figure 11b–g shows the top view, front view, bottom view, upper surface point clouds of the composite material, lower surface point clouds of the composite material, lower surface point clouds of the stringers and isometric view of the 3D ultrasonic inspection results, respectively.

To ensure the rigor of dimensional measurement, the ground truth dimensions of the test piece were obtained using a coordinate measuring machine (CMM) with a measurement uncertainty of ±0.005 mm. The measurement process was carried out under strictly controlled environmental conditions: temperature 20 ± 2 °C, humidity 40–60%, and no vibration. For each key dimension, 10 repeated measurements were performed at evenly distributed measurement points, and the ground truth dimension was calculated as the mean value of these measurements, with its standard uncertainty derived from the standard deviation of the repeated measurements.

Subsequently, the 3D reconstruction point cloud of the composite component was precisely registered with the CMM measurement point cloud using the iterative closest point (ICP) algorithm. Before registration, outliers in both point clouds were eliminated through statistical filtering to improve registration accuracy. The registration residual was controlled within a threshold of 0.02 mm, and the final registration results showed a mean residual error of 0.012 mm and a maximum residual error of 0.018 mm, ensuring the reliability of subsequent dimensional comparison.

It can be seen from the figures that the 3D ultrasonic inspection imaging results are highly consistent with the actual material shape. After accurate registration, the dimensional measurement error is less than 0.1 mm (actual dimensions (ground truth mean value): 400.68 × 300.50 × 40.36 mm; 3D imaging dimensions: 400.76 × 300.58 × 40.42 mm; errors: 0.08 mm, 0.08 mm, and 0.06 mm, respectively). Three stringer debonding damages are detected in Figure 11f, with the debonding lengths of 52.86 mm, 35.02 mm and 33.04 mm, respectively, and the imaging results are completely consistent with the actual spatial distribution of the damages. This indicates that the method proposed in this paper has an excellent overall fitting ability for large-size composite laminated structures and meets the engineering inspection accuracy requirements.

#### 4.4.2. Damage Simulation Specimen of Composite Laminated Panel

**Figure 12:**
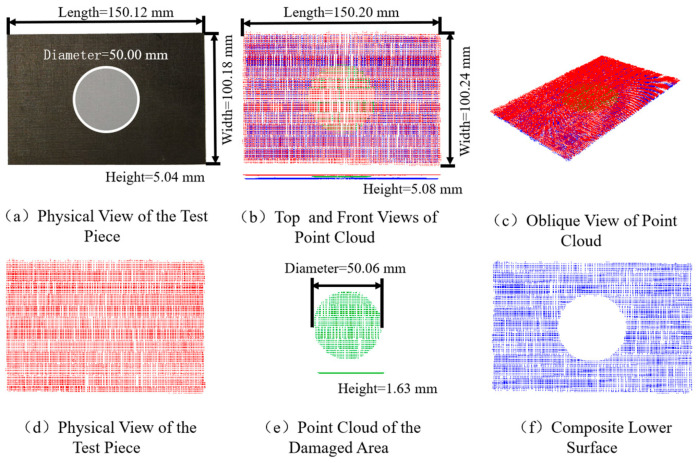
Intelligent Ultrasonic Testing Results of Composite Laminated Plate Damage Simulation Specimen.

We conducted fusion detection experiments on damage-simulated specimens of composite laminates, and obtained the final 3D ultrasonic inspection results using the method proposed in this paper. Figure 12 shows the intelligent ultrasonic detection results of the damage-simulated composite laminate specimen. Specifically, Figure 12a–f present the physical schematic of the specimen, the top and front views of its 3D ultrasonic imaging, an oblique view of the 3D ultrasonic imaging, the point cloud of the upper surface, the point cloud of the damage region, and the point cloud of the lower surface of the specimen, respectively.

It can be observed from the figures that the 3D ultrasonic imaging results are highly consistent with the actual geometry of the material, with a dimensional measurement error of less than 0.1 mm (actual dimensions: 150.12 × 100.18 × 5.04 mm; 3D imaging dimensions: 150.20 × 100.24 × 5.08 mm; corresponding errors: 0.04 mm, 0.06 mm, and 0.04 mm). This demonstrates that the proposed method exhibits excellent overall fitting performance for flat composite plates.

Figure 12e shows the 3D imaging of the circular internal damage in the material, with a measured diameter of 50.06 mm, which differs by only 0.06 mm from the designed actual damage. The imaging morphology is also consistent with the real damage configuration, verifying the effectiveness of 3D ultrasonic imaging for internal damage localization in flat composite laminates.

Figure 12f displays the 3D imaging of the bottom surface of the material, where a circular signal-deficient region appears in the middle. This is because most of the ultrasonic energy is reflected by the circular internal damage, resulting in weakened ultrasonic energy reaching the bottom surface, which is thus removed during signal processing.

By observing Figure 12b,d–f, it can be seen that the robotic arm adopted a lateral scanning strategy with stable scanning speed and uniformly distributed data acquisition.

## 5. Conclusions

Aiming at the bottleneck problems of 3D ultrasonic inspection for aircraft composite laminated structures, including heavy reliance on manual operation, low efficiency, and the inability of traditional robotic arms to adapt to complex curved surface inspection due to the need for preset fixed trajectories, this paper proposes an innovative automated 3D ultrasonic inspection method guided by robotic arm 3D vision. First, an advanced region segmentation algorithm is used to perform visual recognition and region segmentation on composite components; the image segmentation results are mapped to the depth map, and the material surface point cloud is obtained through conversion. Then, voxel downsampling, normal vector calculation, equal interval sampling and trajectory point extraction are carried out based on the material surface point cloud. Subsequently, the robotic arm performs precise movement according to the extracted trajectory points, and the system synchronously records its high-precision spatial pose data and ultrasonic inspection data during the scanning process in real time. Finally, through a proprietary fusion algorithm, 3D reconstruction of spatial coordinates and ultrasonic information is realized to generate intuitive and accurate 3D images of the internal material structure, thereby achieving accurate spatial localization of damages and automated evaluation of structural integrity. This method is intended to completely break away from the reliance on manual experience and provide an effective full-process automated solution for solving the problems of diverse aircraft composite structures and variable inspection conditions. The main conclusions are as follows:

1. The YOLO-Mask model exhibits excellent segmentation performance: Experimental results show that the model effectively improves the accuracy and efficiency of regional segmentation for composite laminated structures through the innovative design of the C3EMA and CARAFE modules; ultimately, the Segm-mAP of the inspection reaches 97.4% with a detection speed of up to 11.7 fps. In addition, the segmented regions can be mapped to the depth map and further converted into material surface point clouds, which provide data for subsequent robotic arm guidance.
2. The 3D point cloud-based scanning trajectory planning for robotic arms is accurate and efficient: This method ultimately realizes the automatic planning of robotic arm ultrasonic scanning trajectories based on the segmented material surface point cloud. By processing the point cloud data and fusing calibration parameters, the system can automatically calculate the six-dimensional pose coordinates of inspection points and drive the robotic arm to complete fully automated ultrasonic inspection operations.
3. The 3D imaging effect is accurate and reliable: Guided by 3D vision, the robotic arm automatically performs high-precision scanning of the material; by combining the end-effector coordinates of the robotic arm with the processed ultrasonic B-scan data, high-precision 3D imaging of the interior of composite laminated structures is realized, with the 3D imaging error being less than 0.1 mm.

## Figures and Tables

**Table 1:** Comparison results of AUC with other models.

Model	dB			Average
	10	15	20	
AE	0.844	0.854	0.849	0.849
VAE	0.889	0.904	0.898	0.897
LVAE	0.973	0.985	0.982	0.980

**Table 2:** Comparison of Yolo-Mask with other modules.

Moudle	Bbox-mAP/%	Segm-mAP/%	fps
Yolo11	93.0	91.2	7.6
Mask R-CNN	94.6	92.6	5.5
DynaMask	96.3	95.8	1.7
Yolo-Mask	98.7	97.4	11.7

**Table 3:** Results of the ablation experiment.

Improvement		Bbox-mAP/%	Segm-mAP/%	fps
C3EMA	CARAFE			
-	-	93.6	92.2	18.6
√	-	95.9	94.6	15.6
-	√	97.2	95.9	13.3
√	√	98.7	97.4	11.7

**Table 4:** End-to-end cycle-time and efficiency analysis of the robotic inspection system.

Size	St1	St2	St3	St4	St5	St6	Time	mm^2^/s
100 × 100	0.82	1.25	0.68	12.34	2.17	1.53	18.79	53.22
300 × 300	0.85	3.42	1.95	41.26	6.83	4.79	59.10	152.28
500 × 500	0.88	5.71	3.24	78.55	11.38	8.62	108.38	230.21

## Data Availability

Data are available upon request from the corresponding authors.
